# Advancing Accuracy in Non-invasive Hemoglobin Estimation: A Comparative Clinical Study of the Performance of the Non-invasive Anemia Detection App (NiADA)

**DOI:** 10.7759/cureus.88435

**Published:** 2025-07-21

**Authors:** Krishanu Banerjee, Tuphan Kanti Dolai, Abhishek Sharma, Ivan Chernukha, Debjeet Das, Vipul Sharma, Mou Nandi

**Affiliations:** 1 Artificial Intelligence, Monere Corporation, Lehi, USA; 2 Hematology, Nil Ratan Sircar (NRS) Medical College and Hospital, Kolkata, IND; 3 Artificial Intelligence, Monere Corporation, Kolkata, IND; 4 Artificial Intelligence, Monere Corporation, Delhi, IND

**Keywords:** anemia, artificial intelligence, hemoglobin monitoring, image processing, non-invasive, smart phone app

## Abstract

Background: Anemia remains a significant global health burden, particularly in low- and middle-income countries. Traditional hemoglobin screening methods are invasive, resource-intensive, and often impractical for large-scale or repeated population-level screening. The Non-invasive Anemia Detection App (NiADA, Monere AI Private Limited, Kolkata, West Bengal, India) provides a smartphone-based, artificial intelligence (AI)-powered alternative for estimating hemoglobin levels using images of the lower palpebral conjunctiva.

Objective: This study aims to evaluate the improvement in accuracy and clinical utility of NiADA version 3 compared to NiADA version 2 in estimating hemoglobin levels and detecting anemia across diverse demographic subgroups in a tertiary care setting.

Materials and methods: This study was conducted at NRS Medical College and Hospital, Kolkata, India, from December 2024 to January 2025. A total of 2,476 participants (ages 2-90 years) were enrolled. Trained personnel captured partial facial images, focusing on the lower eyelid, using Android smartphones running NiADA version 3. The algorithm subsequently extracted the lower palpebral conjunctiva and surrounding scleral regions for automated analysis. Images underwent preprocessing and were analyzed in real time by the AI model. Venous blood samples were collected immediately after image capture in standard ethylenediamine tetraacetic acid anticoagulant tubes, and hemoglobin levels were measured using an automated hematology analyzer. Regression and classification performance were evaluated using Bland-Altman analysis, mean bias, Lin’s concordance correlation coefficient (CCC), and confusion matrices. Subgroup analyses were performed for adult males, adult females, and children.

Results: NiADA exhibited strong agreement with laboratory-measured hemoglobin levels across all demographic subgroups, with Pearson correlation coefficients ranging from 0.81 to 0.86, Lin’s CCC between 0.80 and 0.87, and R² values spanning 0.73 to 0.76. The mean bias remained within ±0.27 g/dL across cohorts. Bland-Altman analysis showed that over 95% of predictions fell within the limits of agreement for children (−2.07 to 2.46 g/dL), females (−2.48 to 2.64 g/dL), and males (−2.51 to 3.05 g/dL). In anemia classification, NiADA achieved the highest accuracy in adult females (88.7%), followed by children (84.4%) and adult males (81.2%). Sensitivity remained consistently high across all groups (≥88%), while specificity ranged from 71.8% to 76.1%.

Conclusions: NiADA version 3 demonstrates strong accuracy and reliability as a non-invasive hemoglobin estimation tool, with performance comparable to that of conventional point-of-care devices. Its smartphone-based, consumable-free workflow makes it particularly well-suited for large-scale screening and longitudinal monitoring in both clinical and community settings. These results support NiADA’s integration into public health initiatives targeting anemia surveillance and prevention.

## Introduction

Anemia remains one of the most pressing global health challenges, affecting approximately 1.92 billion individuals, nearly a quarter of the world’s population, according to both the World Health Organization and the Global Burden of Disease Study 2021 [1]. It disproportionately impacts women of reproductive age, children, and pregnant individuals, particularly in low- and middle-income countries such as India [2]. It has been found to cause multiple health problems, leading to more than 50 million years of healthy lives lost due to disability [3]. The presence of anemia has also been associated with higher mortality and poor disease outcomes [4]. In India alone, programs such as Anemia Mukt Bharat have emphasized the need for large-scale screening and routine monitoring to meet national targets for reducing anemia, particularly among vulnerable populations. However, current screening methods often rely on invasive blood-based testing, which can be resource-intensive, uncomfortable for patients, and logistically challenging to scale in low-resource or field settings. These limitations underscore the need for non-invasive, rapid, and easily deployable solutions such as the Non-invasive Anemia Detection App (NiADA, Monere AI Private Limited, Kolkata, West Bengal, India) [5].

The current gold standard for laboratory estimation of hemoglobin is the cyanmethemoglobin method, also known as the cyanide hemoglobin method, which is used for estimating hemoglobin in venous blood [6]. However, in routine clinical settings, including this study, hemoglobin levels are typically measured using automated hematology analyzers. However, this method is operationally complex and may be challenging to scale in resource-limited or field-based settings due to infrastructure, staffing, and logistical constraints [7]. Most of the currently available methods for hemoglobin estimation, including the gold standard and point-of-care testing (POCT) devices, are invasive, such as HemoCue. These approaches require trained personnel, physical infrastructure, and consumables, all of which create friction in community-level deployment [8]. Moreover, repeated blood pricks discourage participation, particularly among children and pregnant women. An acceptable solution to these problems would be a non-invasive method that will make the process painless, real-time, smooth, and more accessible. Being non-invasive, this method will also reduce the risk of infection, the need for external equipment, the likelihood of exposure to healthcare professionals, and the generation of biomedical waste [9]. These reasons have led to a growing interest in using non-invasive, POCT solutions for hemoglobin estimation in recent years.

To address these challenges, NiADA has been developed. It leverages smartphone imaging of the lower palpebral conjunctiva and advanced artificial intelligence (AI) to estimate blood hemoglobin levels in real-time. This approach offers a cost-effective, non-invasive, and scalable alternative to traditional methods. Earlier clinical validations of NiADA at All India Institute of Medical Sciences, Kalyani [10], and North Bengal Medical College [11] demonstrated promising results, with performance metrics comparable to or better than those of human estimation and specific point-of-care devices, such as HemoCue.

The present study marks the third phase of NiADA’s clinical validation. Our objective is to systematically evaluate the diagnostic accuracy and implementation performance of NiADA version 3 for non-invasive hemoglobin estimation. This includes assessing its predictive reliability across age and gender subgroups, as well as its generalizability across commonly used smartphone devices in real-world clinical settings.

## Materials and methods

Study design and setting

This prospective validation study was conducted at NRS Medical College and Hospital, Kolkata, India, over a period of two months (December 2024 to January 2025). The study received ethical clearance from the Institutional Ethics Committee of NRS Medical College and Hospital (approval number: ECINEW/INST/2023/1862). Written informed consent was obtained from all participants or their legal guardians. All study procedures adhere to the principles outlined in the Declaration of Helsinki and relevant national guidelines on ethics.

Inclusion and Exclusion Criteria

Participants were recruited from the outpatient hematology department at a tertiary care hospital. Recruitment was general and non-selective and did not require prior clinical suspicion of anemia. The inclusion and exclusion criteria are detailed in Table 1. In future versions of NiADA, filters will be implemented, and poor-quality images will not be allowed to be captured.

**Table 1:** Inclusion and exclusion criteria NiADA: Non-invasive Anemia Detection App

Inclusion criteria	Exclusion criteria
Participants aged ≥2 years	Eye conditions that impede clear lower eyelid photography include infectious/allergic conjunctivitis, chronic eyelid conditions (e.g., blepharitis, contact lens-induced papillary conjunctivitis), dry eye/keratoconjunctivitis sicca, and conjunctival scarring/post-surgical changes.
Willingness to participate. Ability to provide informed consent (for adults). For child participants, parental consent was obtained, and age-appropriate assent was sought from children capable of providing it, following institutional ethics protocols	Poor image quality as determined by predefined NiADA filters (e.g., blurred images, over/underexposure, excessive reflection)

Image capture and application workflow

Trained field workers used common smartphones (with a minimum of 4GB RAM and a 12MP camera) running the NiADA application to capture images of the lower eyelid under standardized ambient lighting conditions, specifically 4000K-6500K white light with an intensity of approximately 300-500 lux, as found in typical hospital outpatient settings. Room temperature was maintained between 20°C and 24°C (68°F-75°F) to ensure patient comfort and imaging consistency. In this study, five cell phones were used: one Samsung (SM-A166P, Samsung Electronics Co., Ltd., Gyeonggi-do, South Korea), one Xiaomi (M2007J17I, Xiaomi Incorporated, Beijing, China), and three Vivo (V2055, V2355, V2351, Vivo Mobile Communication Co., Ltd., Guangdong, China) models. For each participant, images were securely uploaded to the NiADA server, where the AI model processed them in real time to estimate hemoglobin levels.

Captured images underwent preprocessing, including contrast and white balance correction, rejection of illumination artifacts using correlated color temperature and brightness histogram analysis, and blurriness filtering through edge-density and Laplacian-based techniques.

Laboratory reference and ground truth

Approximately 2-3 mL of venous blood was collected from each participant immediately after image capture, using standard ethylenediamine tetraacetic acid-coated vacutainer tubes to prevent coagulation. Samples were processed at the hospital’s National Accreditation Board for Testing and Calibration Laboratories (NABL)-accredited central laboratory following institutional standard operating procedures. Hemoglobin levels were measured using automated hematology analyzers (e.g., Sysmex XP-100 or equivalent three-part/five-part models, Sysmex Corporation, Hyōgo Prefecture, Japan), which employ non-cyanide photometric methods consistent with the International Council for Standardization in Haematology recommendations. All instruments were routinely calibrated and underwent daily internal quality control checks. While multiple analyzer models may have been used, all were compliant with NABL standards. Hemoglobin measurements obtained from these analyzers served as the clinical reference standard for model validation.

Study population

The study initially enrolled 2,476 participants, spanning a broad demographic range, including men, women, and children aged 2 to 90 years. After data collection, images were subjected to a two-tier exclusion process. First, images identified as of poor quality (e.g., blurred, improperly exposed, or with excessive glare) by predefined NiADA filters were removed. Subsequently, 205 samples (8.2%) were excluded based on manual physician review, which involved checking for clinical inconsistencies, labeling errors, or unusable metadata (e.g., missing or mismatched hemoglobin values). The final dataset comprised only high-quality, physician-verified image-lab result pairs.

**Figure 1:**
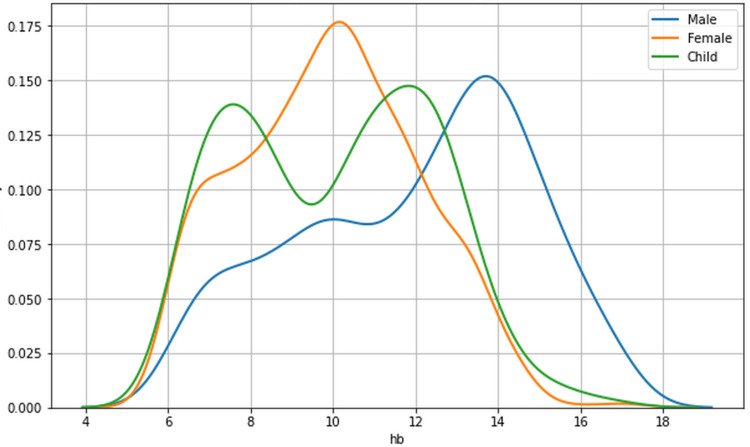
Hemoglobin distribution by gender (population count)

Hemoglobin levels among participants ranged from 6 g/dL to 17 g/dL. Study population summary statistics are presented in Table 2, and hemoglobin distribution by segment in Figure 1.

**Table 2:** Study population statistics summary SD: standard deviation

Segment	Sample size	Age (mean ± SD)	Hemoglobin (g/dL) (mean ± SD)
Female	834	42.88 ± 13.28	9.92 ± 2.15
Male	899	47.41 ± 14.56	11.93 ± 2.82
Child	538	11.04 ± 4.57	10.08 ± 2.40

It was observed that hemoglobin levels in the female population tended to cluster at the lower end of the range. In contrast, the male population showed a peak distribution at higher hemoglobin levels.

Statistical analysis

Statistical analysis was conducted to evaluate the agreement between NiADA-predicted and laboratory-measured hemoglobin levels, both continuous and categorical. The agreement between NiADA and lab results was evaluated using regression and classification analysis. Subgroup analysis was performed for males (age > 19), females (age > 19), and pediatric patients (2 < age < 19). Outliers were defined as samples with a prediction error >2.5 g/dL. The methods and corresponding units or metrics used are summarized in Table 3.

**Table 3:** Statistical methods overview CCC: concordance correlation coefficient, CI: confidence interval, NiADA: Non-invasive Anemia Detection App

Analysis type	Method/metric	Unit/description
Hemoglobin estimation	Laboratory and NiADA-predicted values	g/dL
Regression analysis	Bland–Altman plot	Mean difference, 95% CI
	Coefficient of determination	R² (range: 0 to 1)
	Lin’s CCC	Unitless (range: -1 to 1)
	Pearson correlation coefficient	Unitless (range: -1 to 1)
Classification by cohort	Accuracy	Percentage (%)
	Sensitivity (true positive rate)	Percentage (%)
	Specificity (true negative rate)	Percentage (%)

## Results

Regression analysis

**Figure 2:**
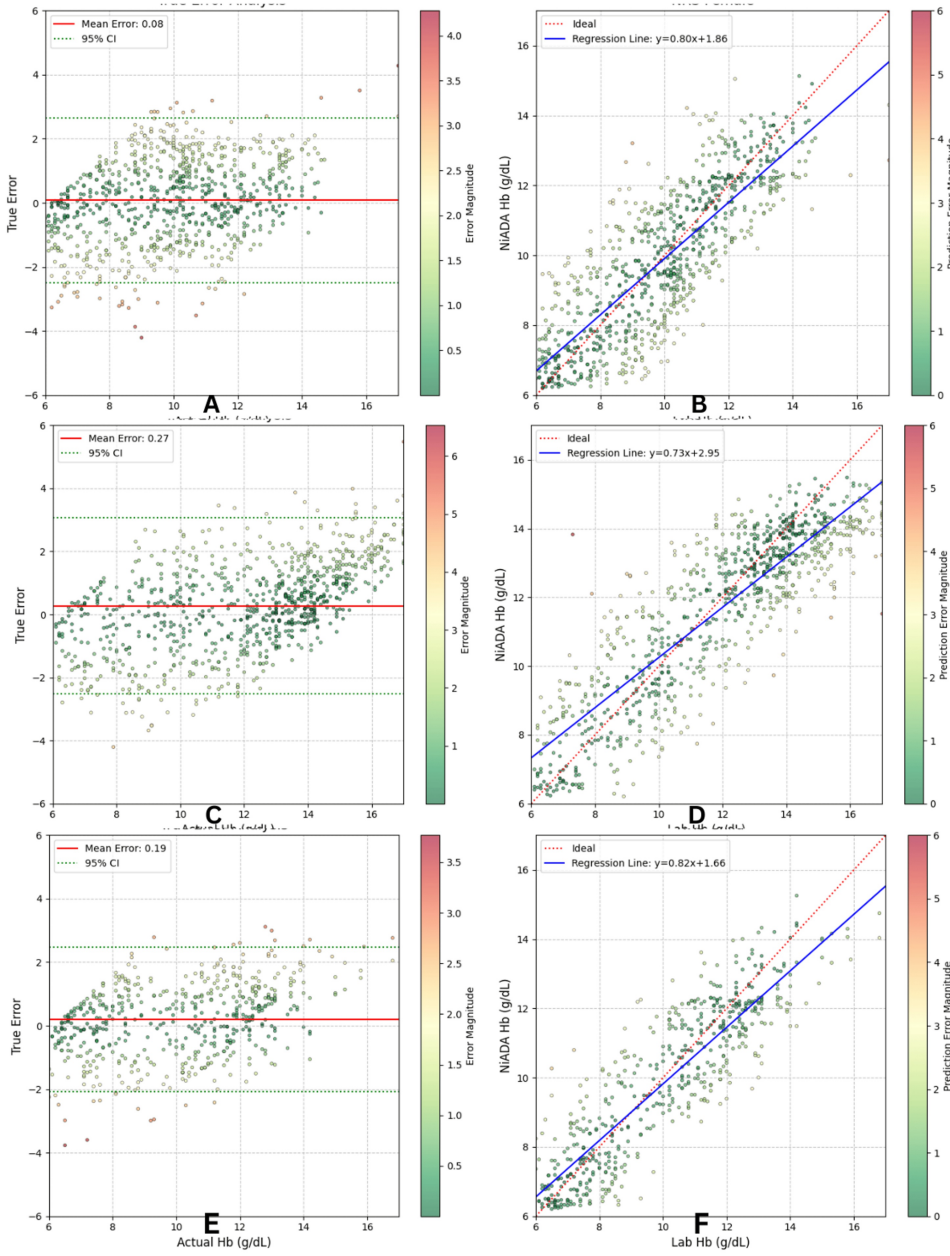
Regression results for child, female and male (A) Top-left: Bland-Altman plot for females. (B) Top-right: lab Hb (g/dL) vs. NiADA (g/dL) prediction for females. (C) Middle-left: Bland-Altman plot for males. (D) Middle-right: lab Hb (g/dL) vs. NiADA (g/dL) prediction for males. (E) Bottom-left: Bland-Altman plot for children. (F) Bottom-right: lab Hb (g/dL) vs. NiADA (g/dL) prediction for child. CI: confidence interval, Hb: hemoglobin, NiADA: Non-invasive Anemia Detection App

Regression analyses were conducted separately for children, adult males (male), and adult females (female) to evaluate the predictive performance of the NiADA version 3 against laboratory hemoglobin values. Figure 2 illustrates the results of the regression analysis. Bland-Altman plots (A, C, E) show the left, and the regression results are shown on the right (B, D, F); the lab analyzer value of hemoglobin (g/dL) is compared with the NiADA prediction (g/dL).

Children

In the pediatric subgroup, NiADA's hemoglobin estimates exhibited a strong linear correlation with laboratory measurements, with a slope of 0.76, indicating consistent proportional agreement across the range of values. The coefficients suggest a slight proportional underestimation at lower hemoglobin ranges, offset by an intercept (+2.34) shift. The Bland-Altman plot for this group showed a mean true error of -0.19 g/dL, indicating minimal systematic bias. The 95% confidence interval (CI) band reveals symmetric dispersion around the mean, with most predictions falling within ±2 g/dL of lab values. Some error clustering was observed near the anemia cutoff threshold (~11 g/dL), likely due to natural variation in palpebral image signal and associated illumination correction challenges in pediatric scans.

Males

In adult males, the regression analysis revealed a shallower slope (0.67) and a higher intercept (+3.84) compared to the pediatric group. This pattern suggests a mild compression in NiADA's predicted hemoglobin values, with slight underestimation at higher levels and overestimation at lower levels. The mean true error was +0.27 g/dL, slightly higher than for children, yet still within clinically acceptable limits. The Bland-Altman plot showed broader error variability, particularly in the 12-14 g/dL range. This may be attributable to higher physiological hemoglobin variability in adult males and stronger skin pigmentation artifacts in this cohort, which could impact the generalization of the AI model.

Females

Among adult females, NiADA demonstrated the most balanced regression performance, characterized by a strong linear relationship (slope of 0.77 and an intercept of +2.42) and minimal bias across the entire hemoglobin range. The mean true error was only +0.08 g/dL, indicating excellent alignment with laboratory values. The error distribution was tight and symmetric, with few significant outliers. The regression line closely followed the identity line, and the variance of prediction errors was notably lower than that of the male cohort. This suggests that the current model architecture is particularly well-calibrated for female palpebral conjunctiva characteristics, possibly due to greater representation in the training dataset and more consistent imaging conditions in this subgroup. The results of the regression analysis are summarized in Table 4.

**Table 4:** Regression summary for adult female, adult male and child * lower limit/upper limit (upper limit = bias + 1.96 * SD and lower limit = bias - 1.96 * SD) CCC: concordance correlation coefficient, LOA: limits of agreement, SD: standard deviation

Group	Mean bias (g/dL)	LOA*	R-square	Pearson r	Lin's CCC
Female	-0.08	-2.48/2.64	0.77	0.81	0.813
Male	-0.27	-2.51/3.05	0.73	0.86	0.84
Children	-0.19	-2.07/2.46	0.76	0.878	0.874

The subgroup-specific regression patterns suggest that NiADA's performance is consistent across populations but can be influenced by subtle differences in skin tone and vascular visibility, conjunctival exposure, image alignment, and natural variation in physiological hemoglobin across age and gender.

In all three cohorts, the model demonstrated high concordance, low mean error, and predictable bias trends that can be corrected via post-regression calibration in future versions. Importantly, no severe outlier patterns were observed, and most predictions fell within ±2 g/dL of lab values, meeting the minimum clinical relevance threshold for non-invasive tools.

To evaluate NiADA's consistency with laboratory hemoglobin estimation, mean hemoglobin values and standard deviations were compared across major demographic subgroups. As shown in Table 5, below, NiADA closely approximates the laboratory mean across all groups, demonstrating minimal bias and high agreement.

**Table 5:** Population statistics comparison NiADA: Non-invasive Anemia Detection App, SD: standard deviation

Groups	Lab (g/dL) (mean ± SD)	NiADA (g/dL) (mean ± SD)
Female	9.92 ± 2.15	9.84 ± 2.13
Male	11.93 ± 2.82	11.66 ± 2.38
Child	10.08 ± 2.4	9.88 ± 2.23

Classification analysis

NiADA exhibited robust classification performance across all demographic subgroups in detecting anemia, using clinically established hemoglobin thresholds of 12 g/dL for adult females, 13 g/dL for adult males, and 11 g/dL for children, as defined by the World Health Organization [12]. In this evaluation, a true positive (TP) was defined as cases where both NiADA and laboratory testing identified hemoglobin levels below the respective thresholds. A true negative (TN) occurred when both NiADA and laboratory values were above these thresholds. False positives (FP) represented instances where NiADA identified a non-anemic individual as anemic. Meanwhile, false negatives (FN) referred to cases in which NiADA failed to identify anemia in individuals with sub-threshold hemoglobin values.

The classification metrics were computed as follows:

\begin{align*} \text{Accuracy} &= \frac{TP + TN}{TP + TN + FP + FN} \\ \text{Sensitivity} &= \frac{TP}{TP + FN} \\ \text{Specificity} &= \frac{TN}{TN + FP} \end{align*}

**Figure 3:**
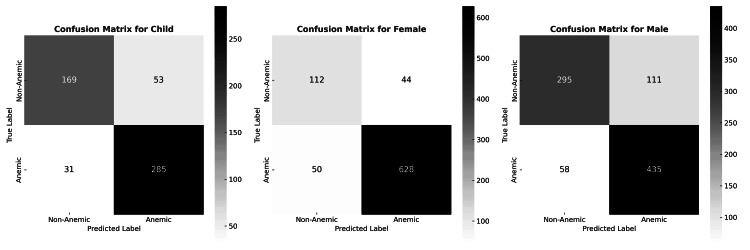
Confusion matrix for child, male and female True label: anemic or non-anemic, classified by lab value. Predicted label: anemic or non-anemic, classified by NiADA. NiADA: Non-invasive Anemia Detection App

NiADA achieved the highest accuracy in adult females (88.7%), along with excellent sensitivity (92.6%) and acceptable specificity (71.8%). In the pediatric cohort, performance remained strong, with an accuracy of 84.4%, a sensitivity of 90.2%, and a specificity of 76.1%, underscoring the model's applicability for children despite known variability in image capture. The lowest overall accuracy was observed in adult males (81.2%), primarily due to a moderate specificity of 72.7%, though sensitivity remained high at 88.2%. These findings indicate that NiADA is well-calibrated for anemia screening across diverse populations. Further optimization, particularly aimed at improving specificity in males, may enhance its precision for broader population-level deployment. Figure 3 shows the confusion matrix, and Table 6 summarizes the classification results by cohort groups.

**Table 6:** Classification summary

	Female	Male	Child
Accuracy	0.887	0.812	0.844
Sensitivity	0.926	0.882	0.902
Specificity	0.718	0.727	0.761

Cell phone variability

**Figure 4:**
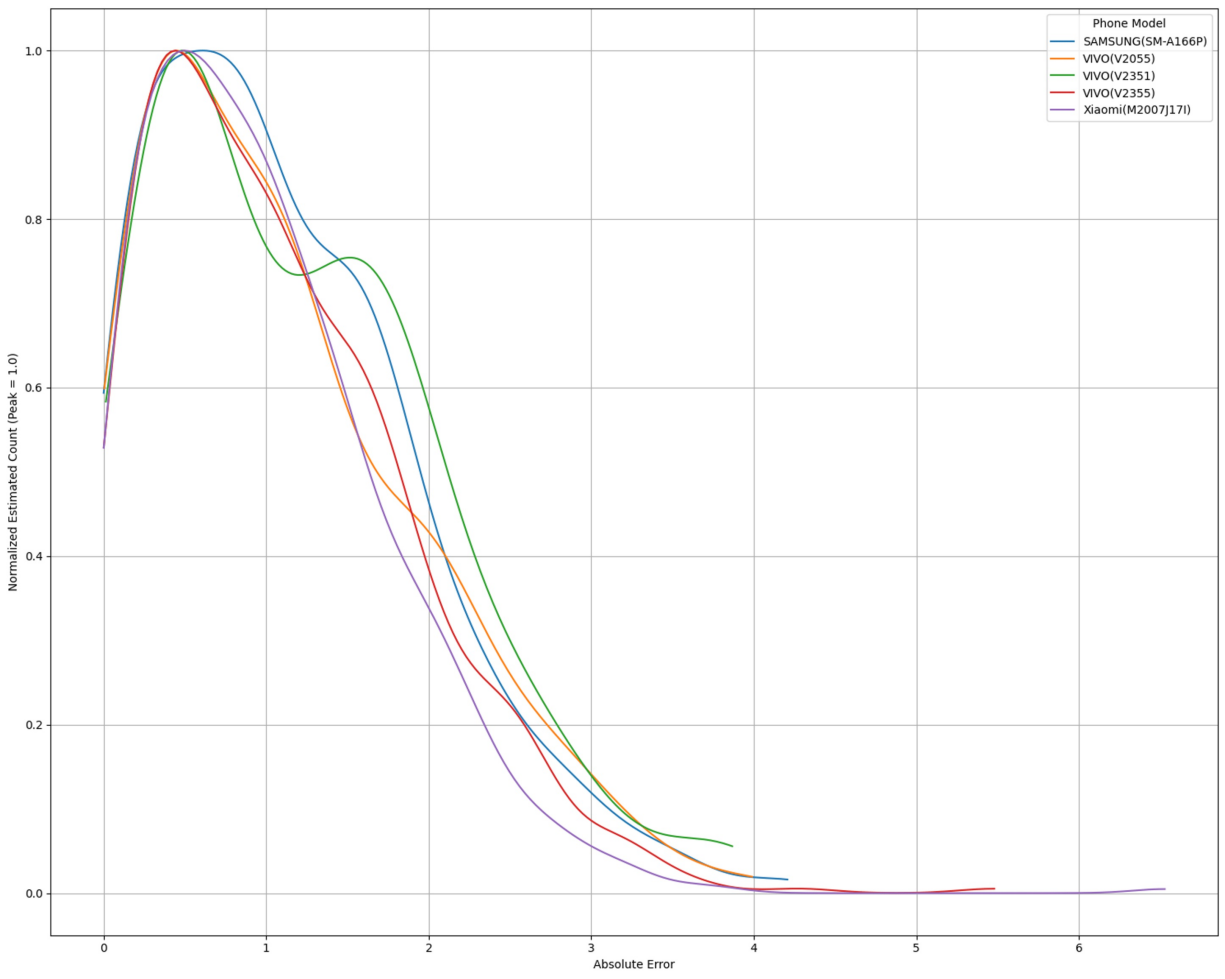
Cell phone variability

Figure 4 illustrates the normalized distributions of absolute prediction errors across five widely used smartphone models: Samsung (SM-A166P), Vivo (V2055), Vivo (V2351), Vivo (V2355), and Xiaomi (M2007J17I). These distributions were smoothed using Gaussian kernel density estimation, with the bandwidth determined via Silverman's rule of thumb. No outliers were excluded prior to smoothing or normalization. The resulting curves were then scaled to a peak value of 1.0, allowing for direct visual comparison of the distribution shape and spread across devices. Minor variations in performance are observed among devices, likely due to differences in their integrated signal processing (ISP) hardware.

All models display a similarly skewed distribution, with most errors concentrated below 0.5 g/dL, indicating consistent predictive accuracy across all devices. However, subtle inter-device differences emerge: Samsung (SM-A166P) and Vivo (V2055) exhibit sharper peaks and narrower tails, suggesting tighter error bounds, while Xiaomi (M2007J17I) and Vivo (V2355) show broader curves, implying increased variance and greater sensitivity to ISP-induced noise.

These findings reinforce the model's robustness across diverse smartphone platforms while also emphasizing the need for ongoing validation across varied hardware configurations, particularly in clinical or field-based implementations.

## Discussion

The third phase of NiADA's clinical validation at NRS Medical College and Hospital marks a substantial advancement in non-invasive hemoglobin estimation, with Lin's concordance correlation coefficient (CCC) increasing by 10-12% and classification accuracy improving by 15-17% compared to version 2. These gains reinforce NiADA's trajectory from a proof-of-concept tool to a clinically viable digital diagnostic. Compared to earlier studies conducted at the All India Institute of Medical Sciences, Kalyani, and North Bengal Medical College, where NiADA versions 1 [10] and 2 [11] exhibited promising correlation and feasibility, the current study showcases significant performance enhancement with the NiADA version 3 algorithm.

In the prior study at the All India Institute of Medical Sciences, Kalyani, NiADA demonstrated a Pearson correlation of 0.6 and Lin's CCC of 0.59, with relatively higher prediction variability near anemia thresholds [10]. Notably, the study was conducted on a smaller sample (~500 participants), whereas the current validation at NRS Medical College and Hospital involved a substantially larger and more diverse cohort (n=2,476). The present NRS validation shows that NiADA has matured significantly in both technical implementation and clinical performance. Regression agreement is consistently high across adult females, adult males, and pediatric cohorts, with Pearson correlations improving to above 0.86 in males and children, and to 0.81 in females. Lin's CCC was also consistently high (0.8-0.86), underscoring the model's alignment with lab values. The R2 value is consistently high, ranging from 0.73 to 0.77. These metrics reflect more consistent image preprocessing, robust outlier handling, and enhanced calibration for diverse demographic groups. NiADA version 3 achieved an overall accuracy of 88.7% for adult females, 84.4% for children, and 81.2% for adult males, representing an average increase of 15-17% over earlier versions. Sensitivity remained particularly strong (≥90% in children and females), supporting the tool's utility in frontline screening. While specificity in males was comparatively lower (72.7%), this may be improved in future iterations through expanded training on male samples.

The performance metrics of NiADA version 3 favorably compare with other established non-invasive hemoglobin assessment tools. For instance, AnemoCheck (Sanguina, Inc., Peachtree Corners, GA, USA) demonstrates limits of agreement (LOA) of ±4.43 g/dL in adults and ±3.54 g/dL in children [13]. Meanwhile, Astrim Fit (Sysmex Corp., Hyōgo Prefecture, Japan) shows LOA of −3.65 to +3.99 g/dL and mean bias of 0.17 ± 1.95 g/dL [14]. NiADA version 3, by contrast, achieves a narrower LOA range of −2.64 to +2.86 g/dL, indicating reduced prediction error and enhanced agreement with laboratory standards. Devices like the Radical-7 (Masimo Corp., Irvine, California, USA) [15] (LOA = -2.30, 1.72 g/dL) and EzeCHeck (EzeRx Health Tech Pvt. Ltd., Bhubaneswar, India) [16] show comparable performance but require proprietary hardware and are cost-intensive. NiADA's key advantage lies in its deployment feasibility, utilizing only smartphone infrastructure and offering a low-cost, scalable solution that is independent of invasive procedures or consumables.

Hemoglobin estimation through eyelid color with different custom state-of-the-art algorithms has also achieved reasonable success. A study at Brown University with 202 patients achieved 72% accuracy [17]. TouchHb is another company that attempted to use a separate camera, but the results showed very poor performance [18]. Ghosal et al. listed some promising approaches that show a true error lower than ±3 [19]. But most of them have been tested on very low sample sizes. NiADA has been trained on more than 70,000 samples and tested in a study involving over 2,000 samples, demonstrating the robustness of the findings, reducing variance, and increasing confidence in performance metrics across heterogeneous populations.

Although NiADA version 3 demonstrates substantial performance improvements over its previous version, several limitations remain. First, the system currently lacks an automated mechanism to reject poor-quality images at the point of capture, which can negatively impact prediction accuracy. Second, performance metrics for male patients are consistently lower compared to those for females and children. This disparity is likely attributable to the higher hemoglobin range typically observed in males, particularly above 14 g/dL, at which point NiADA's predictive performance begins to decline. Additionally, the specificity of NiADA ranges from 71% to 76%, indicating room for improvement. While high sensitivity is more critical for a screening tool such as NiADA, improving specificity would further strengthen its utility and reduce false positives in large-scale deployments. While NiADA demonstrated consistent performance across multiple smartphone models, minor variability in prediction error was observed. This may be attributable to device-specific differences in ISP, such as sharpness and white balance. Smartphone model was not explicitly controlled for in the current analysis, and future work may benefit from incorporating device-specific calibration or normalization strategies.

NiADA offers a distinct advantage through its non-invasive, infrastructure-light, and AI-powered platform, which can operate entirely on smartphones without consumables or blood draws. Moreover, NiADA's capacity to output both continuous (regression) and categorical (classification) hemoglobin estimates positions it uniquely for integration into public health screening and longitudinal monitoring programs.

## Conclusions

NiADA version 3 represents a clinically significant advancement in non-invasive hemoglobin estimation, demonstrating strong concordance with laboratory standards and reliable classification performance across diverse age and gender groups. Validated in a large and demographically diverse cohort in a tertiary care setting, it has demonstrated robust accuracy, particularly in females and children, as well as generalizability across various smartphone devices, positioning it as a practical tool for both clinical and community-based screening. Compared to earlier versions, NiADA version 3 offers improved speed, predictive accuracy, and operational reliability, making it suitable for scalable anemia surveillance. To support broader population-level rollout, future enhancements should prioritize real-time image quality control and performance optimization in higher hemoglobin ranges, especially in males. Additional features such as adaptive thresholding, longitudinal tracking, and device-level calibration may further expand NiADA’s utility in national public health programs.
